# Retraction: Extraction of anthocyanins from purple sweet potato: evaluation of anti-inflammatory effects in a rheumatoid arthritis animal model, mechanistic studies on inflammatory cells, and development of exosome-based delivery for enhanced targeting

**DOI:** 10.3389/fimmu.2026.1839536

**Published:** 2026-04-01

**Authors:** Frontiers Editorial Office

The journal retracts the June 09–2025 article cited above.

Following publication, concerns were raised regarding the integrity of the images in the published figures. An investigation was conducted in accordance with Frontiers’ policies and with the collaboration of the authors. It was concluded that the findings and conclusions of the article were unreliable, and the article has been retracted.

This retraction was approved by the Chief Editors of Frontiers in Immunology and the Chief Executive Editor of Frontiers. The authors were informed of this retraction and agree with the decision.

